# Platelet Transfusions: Current Practices and Emerging Alternatives in the United States

**DOI:** 10.3390/life15060985

**Published:** 2025-06-19

**Authors:** Mark Friedman, Victoria Costa, Behnam Rafiee, Timothy Hilbert, Mansab Jafri, Ding Wen Wu

**Affiliations:** 1 Department of Pathology, New York University Grossman Long Island School of Medicine, Mineola, NY 11501, USA; mark.friedman@nyulangone.org (M.F.);; 2 Department of Pathology, New York University Grossman School of Medicine, New York, NY 10016, USA

**Keywords:** platelets, transfusion, pathogen reduction (PR), platelet additive solution (PAS), large-volume delayed sampling (LVDS)

## Abstract

Platelet transfusions are a cornerstone of hemorrhage management in patients with thrombocytopenia or platelet dysfunction, yet their indications and dosing are largely based on expert opinion and low-quality evidence. This review offers a timely and comprehensive analysis of platelet transfusion practices in the United States (U.S.), uniquely integrating clinical evidence, such as the pivotal PLADO trial, with emerging technological advancements. Using a holistic approach, this manuscript addresses not only conventional practices (such as dosing standards and storage methods), but also cutting-edge alternatives like cold-stored and freeze-dried platelets, pathogen reduction technologies, and synthetic platelet substitutes. By juxtaposing U.S. practices with international standards, it highlights inefficiencies in dosing and supply management, proposing actionable solutions like lower-dose transfusions and diversified platelet inventories. Furthermore, the manuscript’s exploration of whole blood-derived platelets and the ethical debate surrounding paid donors adds a forward-looking perspective. By examining these innovations alongside strategies to optimize supply, this work aims to provide a comprehensive overview of how transfusion medicine is adapting to meet clinical and logistical demands.

## 1. Introduction

The lifesaving role of platelet transfusions in hemorrhage management in cancer patients was first recognized in 1961. Today, platelets are routinely administered either therapeutically to control bleeding or, more often, prophylactically to prevent hemorrhage in patients with thrombocytopenia or platelet dysfunction, although the indications are primarily based on expert opinion and low-quality evidence. The standard platelet dose for adult transfusions in the United States (U.S.) is considerably higher than in most European nations and Canada. However, evidence, including findings from the pivotal PLADO trial, has prompted a reevaluation of U.S. dosing standards. Other concerns surrounding platelet transfusions include bacterial contamination leading to septic transfusion reactions, a result of room-temperature (RT) storage, and the adequacy of the platelet supply, a result of a decreasing donor pool. This review examines current platelet transfusion practices, explores alternatives such as cold-stored and freeze-dried platelets, and discusses synthetic substitutes alongside strategies to optimize supply and utilization.

### 1.1. Platelet Transfusion Indications

Platelets are commonly transfused in the U.S. to manage thrombocytopenic patients either with bleeding or, more often, prophylactically in nonbleeding patients [1]. Platelets are also transfused to patients who have platelet dysfunction, most often a result of antiplatelet medication such as aspirin [1]. In healthy persons, circulating platelets typically range from 150–450,000/µL. While a platelet count of 10,000/µL has been widely adopted as a safe threshold for stable, nonbleeding patients, guideline indications for platelet transfusions in other clinical situations commonly set by hospital facilities based upon published recommendations, such as 50,000/µL in patients who are bleeding or prior to surgical procedures and 100,000/µL prior to neurosurgical procedures, are primarily based upon expert opinion and low-quality evidence [1,2]. Newly published clinical practice guidelines as of 29 May 2025, jointly by the Association for the Advancement of Blood and Biotherapies (AABB) and the International Collaboration for the Transfusion Medicine Guidelines (ICTMG), include expanded recommendations with a focus on more restrictive transfusion practices than the previously published AABB guidelines from 2015 [2,3]. For example, the updated guidelines recommend platelet transfusion for patients undergoing lumbar puncture when the platelet count drops below 20,000/µL in comparison to the previous recommendation of less than 50,000/µL and recommends against platelet transfusion for adults with nonoperative intracranial hemorrhage with platelet counts greater than 100,000/µL, including those receiving antiplatelet medication [2,3]. Nevertheless, these updated recommendations are also primarily based upon low-quality evidence.

### 1.2. Conventional Preparation and Storage of Platelet Products

Platelet components are prepared from either whole blood (WB) or apheresis collections. WB-derived platelets refer to platelet concentrates that are separated out from a WB collection (see the section below for more details on WB-derived platelets). The method of separation differs in the U.S. versus Europe and Canada. In the former, platelets are separated using the platelet-rich plasma (PRP) method whereby soft spin centrifugation precedes a hard spin, increasing red blood cell (RBC) recovery at the expense of plasma recovery [4]. In the latter, platelets are separated via the buffy coat (BC) method, in which hard spin centrifugation precedes a soft spin, resulting in less platelet activation due to the protective effect of cellular components (RBCs and white blood cells [WBCs]) against platelet contact with the container [5]. Activation of platelets in the PRP collection method results in platelet loss during storage. Although conversion from the PRP to the BC method of WB-derived platelet separation is possible in the U.S., there are regulatory barriers to overcome, and apheresis platelets are preferred, accounting for 90–95% of platelet concentrates that are transfused in the U.S. Apheresis platelets are collected from one donor (whereas WB-derived platelets are typically pooled together as a pack of five units), whereby WB is passed through the apheresis device and centrifuged to separate platelets, which are diverted to the collection container along with some plasma, and the remaining WB components (i.e., RBCs, WBCs, and plasma) are returned to the patient. The major reasons why apheresis platelets are preferred in the U.S. are due to their higher platelet yields, reduced donor exposure (i.e., one versus five donor units), and reduced bacterial contamination risk [6].

Both WB-derived and apheresis platelets are suspended in plasma and stored at RT (20–24 °C) with continuous gentle agitation for 5–7 days, depending on the storage container and method of bacterial detection used, as approved by the FDA. RT storage is the major reason for the increased risk of bacterial contamination of platelet concentrates. As a result of the risk of bacterial contamination and consequent septic reactions in recipients, the AABB enacted requirements in March 2004 as part of their Standards that blood banks or transfusion services have methods to limit and detect bacterial contamination of all platelet components [7]. At the time, the prevalence of bacterial contamination of platelet concentrates was estimated to range up to 1 per 3000 units, with clinical sepsis estimated to be 1 per 20,000 platelet transfusions and septic transfusion fatalities in 1 per 60,000 transfusions [7]. In comparison, RBC bacterial contamination rates were estimated at 1 per 500,000 units [7]. However, the above estimates could be skewed by reactions that are classified as febrile nonhemolytic reactions because they were not recognized as being caused by a contaminated platelet unit [7]. In addition, reports to the FDA from 2001–2003 showed that 14.1% of all transfusion-associated fatalities were caused by septic reactions, though the fatality rate may have been three- to four-fold higher given underrecognition and underreporting [8]. Strategies recommended to limit and detect bacteria in platelet collections included the use of better methods to disinfect donor skin (i.e., use of chlorhexidine, a more efficacious antiseptic agent than povidone-iodine), the use of a diversion pouch to prevent the initial 15–30 mL of blood collection and a skin core from entering into the main collection container, and culture-based methods to detect bacteria in platelet concentrates [7,8,9,10]. In 2019, the FDA issued a guidance document listing different one- and two-step methods for bacterial risk control strategies for enhanced safety of platelet collections [6]. The guidance document included enhanced methods of bacterial detection, such as the large-volume delayed sampling culture-based method and rapid bacterial detection (Pan Genera Detection [PGD] test [Verax, Worcester, MA, USA]), and pathogen reduction (PR) technology (Intercept^®^ Blood System, Cerus, Concord, CA, USA) [6,11].

### 1.3. Platelet Dosing

**Figure 1:**
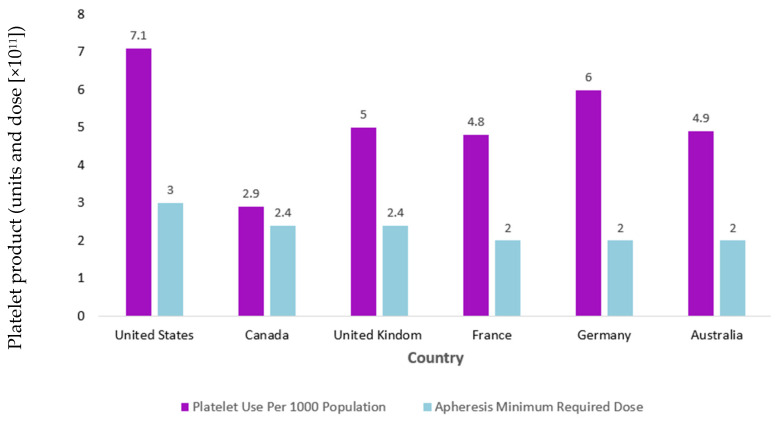
Comparison of international platelet use per 1000 population vs. apheresis minimum required dose. Y-axis label corrected to indicated units and dose (×10^11^), with units in violet and dose in light blue. Chart illustrates that the United States not only transfuses more platelets per capita than other nations but also transfuses platelets at higher doses [19].

In addition, U.S. regulations mandate higher minimum platelet yields per unit compared to Europe and Canada, raising questions about utilization efficiency amid donor shortages [1]. The platelet standard dose set by the U.S. Food and Drug Administration (FDA) in 1972, ≥3.0 × 10^11^ platelets/unit, was historically derived from the average number of platelets in a pool of six whole-blood-derived platelet concentrates rather than on clinical evidence [1]. Meanwhile, standard doses set in many European nations and in Canada are lower, ranging from 2.0 to 2.5 × 10^11^ platelets/unit, without apparent clinical evidence of increased bleeding risk [1]. In support of lowering the U.S. standard platelet dose, the PLADO trial demonstrated that low-dose prophylactic transfusions (median 2.1 × 10^11^ platelets) were non-inferior to standard or high doses in preventing bleeding in thrombocytopenic (platelets < 10,000/μL) hematology–oncology patients [12]. PLADO, published in 2010, was pivotal at the time in that it challenged earlier studies supporting higher platelet doses as well as countermanded the findings of the SToP trial, which, in fact, was stopped early owing to a higher grade 4 bleeding rate in the low-dose study arm [12,13,14,15,16]. The PLADO authors attributed lower rates of severe bleeding associated with low-dose platelet transfusions than found in StoP to the fact that they adjusted the platelet dose for body surface area (BSA), whereas the same dose range was given in SToP without regard to BSA [12,16]. Nevertheless, the results of PLADO were not generalizable to patient populations outside of hospitalized patients with hematological malignancies or solid tumors and hypoproliferative thrombocytopenia that were included in the study, nor could the data be generalizable to younger populations since a subset analysis from the PLADO trial data showed higher rates of bleeding in children [17,18]. Despite these evident limitations, Benjamin et al. [19] proposed lowering the U.S. standard dose to ≥2.5 × 10^11^ platelets in their 2019 commentary, aligning more closely with European and Canadian standards. Lowering the dose, they argued, would increase the production of platelets by up to 23% without altering collection procedures due to increased split unit rates (i.e., collected units from a donor that can be divided into two or three units, depending on the collection yield). This would alleviate chronic shortages of platelet products that occur due to the short allowable shelf life of platelet products (5–7 days, due to RT storage conditions which increase risk of bacterial contamination), high platelet use in the U.S., which has the highest per capita use of platelet transfusions in the world (approximately 2 million platelets transfusions per year), and difficulty in recruiting eligible volunteer donors [19]. In addition, lowering the dose standard would align with platelet product processing methods that are employed to reduce the risk of bacterial contamination and septic reactions but that may result in lower collection yields due to platelet loss during processing [19]. Figure 1 illustrates the platelet use per 1000 population versus apheresis minimum required dose across different nations.

## 2. Whole-Blood-Derived Platelets

While advances in apheresis technology have been notable, with declines in the apheresis donor population and continued post-coronavirus 2019 (COVID-19) pandemic blood supply challenges, interest in the use of WB-derived platelets to supplement the current platelet inventory has emerged [20,21]. Historically, concerns regarding WB-derived platelets included lower corrected count increments (CCIs) and higher rates of bacterial contamination [22]. Nevertheless, newer studies are more supportive of the use of WB-derived platelets. A secondary analysis of platelet transfusions given in a prospective randomized platelet dose study, which included 1272 platelet-transfused hematology–oncology patients, showed that platelet increments were generally higher for transfusions of apheresis platelets, ABO-identical platelets, and platelets stored for 3 days versus those stored for 4 to 5 days, but did not affect the time to occurrence of ≥grade 2 bleeding [23]. This significant finding demonstrated that a higher CCI does not translate into an improved hemostatic outcome. In addition, although WB-derived platelets expose recipients to more donors per adult dose, the data is conflicting in regard to bacterial contamination rates, as some studies have shown equivalent rates between both products while some have shown higher rates with WB-derived platelets, perhaps attributed to effects of routine bacterial testing in more recent studies [11,22,24,25]. The development of pre-pooled (i.e., pooled using sterile technique in the blood center), bacterial-tested platelets (Acrodose^TM^ PL Systems, Haemonetics Corp., Braintree, MA, USA) has been another driver of increased interest in use of WB-derived platelets, with one center reporting at least a comparable response to Acrodose platelets versus apheresis platelets in their oncology patients (including bone marrow transplant patients) with a more robust response to the former in acute leukemia patients [26]. For other risks, including HLA alloimmunization, anti-D alloimmunization, transfusion-related acute lung injury (TRALI), and risk of other adverse reactions, studies have found the risks to be equivalent between both platelet products [22]. Taken altogether, these platelet products can be considered clinically equivalent, and therefore, it would be reasonable to have a mix of products in inventory to help mitigate platelet shortages around the U.S. According to an online survey distributed to medical directors of the 47 America’s Blood Centers (ABC) agencies, 70% of the respondents agreed or agreed strongly that WB-derived platelets and apheresis platelets were clinically equivalent and indicated that the main barrier to the former product implementation was logistic or inventory management issues followed by bacterial contamination risk mitigation [27]. However, 49% of respondents indicated that they are considering producing WB-derived platelets to mitigate shortages but would consider such action in a variety of circumstances, including increasing customer demand, increasing reimbursement, and worsening platelet shortages [28].

## 3. Pathogen Reduction Technology

PR technology, as noted above, is one of the strategies recognized in the FDA guidance document as an accepted method of bacterial contamination risk mitigation [6]. The technology involves the addition of a photoactivating compound to an apheresis platelet unit followed by ultraviolet (UV) irradiation of the product (i.e., UVA or UVB) or direct UVC exposure without the addition of a photosensitizer. In the U.S., the FDA has approved Intercept^®^ Blood System (containing amotosalen [ synthetic psoralen] as the photoactive compound), although in Europe, Mirasol^TM^ (a riboflavin [vitamin B2] photoactivating compound) (Terumo BCT, Lakewood, CO, USA) and Theraflex^®^ UV (Maco Pharma, Mouvaux, France) are also approved PR systems [28,29,30]. While both Intercept and Mirasol platelet products were found to be equivocal in an observational study of massive transfusion patients receiving PR platelets, the Mirasol group required more frequent platelet transfusions, showing evidence of increased consumption (i.e., lower CCIs) perhaps related to a higher platelet activation state, which is, nevertheless, a concern with all PR technology platelets [31,32,33]. Yet, a systematic review found that all three PR technologies (i.e., Intercept, Mirasol, and Theraflex) did not increase bleeding events in comparison to standard platelets, although it confirmed a greater frequency of platelet transfusions and also implicated an increase in platelet refractoriness and alloimmunization associated with PR platelets [32]. On the other hand, pathogen inactivation for Intercept has been reported to be more efficient (i.e., greater inactivation capability) than Mirasol for both bacteria and encapsulated viruses [34,35]. Meanwhile, the Theraflex PR system has been found to be effective against bacteria and protozoa (e.g., Trypanosoma, Leishmania, and Babesia) but is limited in its efficacy against human immunodeficiency virus (HIV), demonstrating only a one-log reduction [36]. Finally, PR technologies offer an equivalent or superior method to inactivate T lymphocytes, and thus are effective in the prevention of transfusion-associated graft-versus-host disease (TA-GVHD), a fatal complication of transfusion in susceptible immunosuppressed recipients (e.g., patients with hematologic malignancies or who have received stem cell transplants) of cellular blood products, including RBC and platelet transfusions [37]. Nevertheless, conversion to PR platelets in the U.S. has been slow to develop, partly due to supply availability but also because of the increased cost associated with implementation as well as the fact that the maximum potential of PR platelets has not yet been realized (e.g., PR platelets are still only approved for 5 days’ storage by the FDA) [38].

## 4. Platelet Additive Solution

PAS is a crystalloid nutrient medium that can replace approximately 60% of the plasma in platelet storage. Compared to plasma-suspended platelets, PAS has the advantage of reduced isoantibody titers (i.e., anti-A and anti-B) in non-group AB donor platelets, lowering the risk of RBC hemolysis and reducing exposure to donor plasma proteins that may trigger allergic transfusion reactions, including more severe anaphylactic reactions, in recipients [39]. In addition, PAS platelets are associated with a lower risk of transfusion-related acute lung injury (TRALI) reactions, a transfusion complication most commonly caused by donor human leukocyte antigen (HLA) antibodies, in buffy coat WB-derived platelets but not apheresis platelets [39]. There are a number of PAS formulations, but currently, only PAS-C (acetate, phosphate) and PAS-F (acetate, potassium, magnesium), are approved for use in the U.S. [39]. PAS may also play a role in mitigating storage lesions caused by pathogen inactivation systems as well as cold storage (discussed below) and may extend platelet concentrate shelf life up to 13 days, although this has not yet been approved by the FDA [39]. Like PR platelets, platelets in PAS may have reduced CCIs, thus increasing the frequency of platelet transfusions [39].

## 5. Cold-Stored Platelets

The FDA established standards for platelets that included cold storage at 1–6 °C with a dating period of 72 h in 1975 [40]. Although CS platelets were commonly used in the 1970s, RT storage became favorable due to the higher recovery and longer survival in circulation in transfused patients. Yet, interest in use of CS platelets has re-emerged, driven by policies to reduce the risk of bacterial contamination associated with RT platelets as well as the understanding that CS platelets take on an “activated” profile, demonstrating greater aggregation in response to agonists, higher clot strength in viscoelastic testing, and improved adhesion under flow compared to RT platelets, making them potentially more suitable for use in trauma and massive bleeding. Furthermore, CS platelets may have longer dating periods and would not require continuous agitation, making them advantageous for use in the pre-hospital and rural settings where maintaining platelet availability may present logistical challenges. Nevertheless, since the FDA has not cleared or approved any blood collection, processing, or storage systems for the manufacture of CS platelets, production has been contingent upon the issuance of exceptions or alternative procedures to the FDA regulations. However, the FDA has recently addressed this by issuance of a guidance document in June 2023 allowing establishments to store apheresis platelets at 1–6 °C for up to 14 days with or without agitation for the treatment of active bleeding when conventional platelets are not available [40].

While much has been published in regard to CS platelets with respect to in vitro function, there is a paucity of data reporting in vivo recovery and function. One review reporting on available data on CS platelet transfusions in humans, including reports dating back to 1964, confirmed what was clinically suspected: that RT platelets consistently had longer circulation times, improving the effectiveness of prophylactic transfusions at the expense of a shorter storage duration owing to the increased risk of bacterial contamination [41]. Meanwhile, the review cautioned that the hemostatic effect of CS platelets is probably very short, possibly lasting only 1 to 2 h, despite circulation times lasting up to 24 h. The authors also noted that in vivo recovery and function of CS platelets could be affected by type of additive solution (i.e., PAS platelets were associated with lower recovery than plasma-suspended platelets), collection type (i.e., older studies prepared platelet concentrates from whole blood versus the apheresis collections in more contemporary studies), and bag types (i.e., older studies used storage bags made of plastic PL 146 versus the polyvinyl chloride in some newer studies which influences oxygen dissociation, pH changes, and platelet survival).

Published experience from a single institution in the U.S., which instituted the use of CS platelets during the peak of the COVID-19 pandemic to mitigate platelet wastage during a time of scarce inventory, reported favorable outcomes (i.e., adequate hemostasis and no overt signal for patient harm associated with CS platelet transfusions), though the report only involved a small sample size (61 CS patients transfused to 40 bleeding patients) [42].

A randomized, single-center pilot trial in Norway compared CS to RT platelets in adult patients undergoing complex cardiothoracic surgery [43]. Platelet storage was limited to 7 days in stage I of the study but extended to 8 to 14 days for CS platelets in the single-arm stage II part of the study. Study enrollment was small, with 25 participants for each arm (i.e., CS and RT) in stage I and 15 participants in stage II. The study was performed with intention-to-treat analysis with a smaller subset post-hoc analysis and with chest tube drain output as the primary outcome and secondary outcomes of platelet function measured by multiple electrode impedance aggregometry after platelet transfusion, blood cell counts, conventional coagulation tests, blood usage, and hemostatic viscoelastic assays until the following postoperative morning. The study supported the feasibility of CS platelets stored for up to 14 days, showing no significant differences in the total blood usage, number of adverse events, length of stay in intensive care, and 28-day mortality between the three study arms in the stage I and II patient groups.

## 6. Cryopreserved and Freeze-Dried (Lyophilized) Platelets

Cryopreserved platelets (CPPs), which date back to the 1950s, consist of adding 6% dimethyl sulfoxide (DMSO) to platelets, concentrating the platelets, and snap freezing them to prevent the formation of ice crystals [44]. CPPs are frozen at −80 °C for 3 years and require thawing and resuspension for use. Cryopreservation causes a change in the structure and shape of the platelets, causing increased expression of phosphatidylserine and P-selectin on the outer membrane that regulates the procoagulant activity of platelets [44,45]. In a phase 1 randomized controlled clinical study, the safety and efficacy of transfusing CPPs in a dose-escalation manner to 28 patients with thrombocytopenia who had active bleeding showed that 58% of the patients receiving CPPs had improved bleeding scores compared with 50% of the patients receiving standard RT platelets and that bleeding also improved in 43% of the patients with World Health Organization (WHO) grade 4 intracranial hemorrhage transfused with CPPs despite lower platelet count increments in the CPP group [46]. However, like other randomized controlled trials involving CPPs, the difference was not statistically significant. Additional trials evaluating CPPs are ongoing.

Freeze-dried platelets (FDPs) are produced by adding trehalose disaccharide or paraformaldehyde to the platelets, which helps preserve platelet quality during processing [44]. FDPs can be stored at RT for 3 years and require reconstitution in sterile water for use. Just as CPPs show increased expression of phosphatidylserine, FDPs do as well. Ohanian et al. [46] performed an open-label phase 1 study of single doses of allogeneic Thrombosomes (i.e., freeze-dried group O platelets loaded with trehalose) at three dose levels in three cohorts, each consisting of eight patients who had hematologic malignancies, thrombocytopenia, and bleeding, to assess the safety and potential efficacy of FDPs. Adverse events, dose-limiting toxicities (DLTs), WHO bleeding scores, and hematology values were assessed. No DLTs were reported. The WHO scores of 22 patients who were actively bleeding at 27 sites at baseline either improved (*n* = 17 [63%]) or stabilized (*n* = 10 [37%]) through day 6. Twenty-four hours after infusion, 12 patients (50%) had a clinically significant platelet count increase. Of the eight patients who received no platelet transfusions for 6 days after Thrombosomes infusion, five had a clinically significant increase in platelet count of ≥5000/μL and two had platelet count normalization. Thrombosome doses up to 3.78 × 10^8^ particles/kg demonstrated safety in 24 bleeding, thrombocytopenic patients with hematological malignancies. Based on their results, these investigators concluded that Thrombosomes may represent an alternative to conventional platelets to treat bleeding.

## 7. Platelet Substitutes

There has been significant interest in developing synthetic platelet substitutes to circumvent the logistical issues and concerns surrounding allogeneic platelet transfusion [47,48]. Research on synthetic platelet substitutes has been ongoing since the 1950s. Platelet substitutes would preferably have the following characteristics: effective hemostasis with an appropriate duration of action, no immunogenicity, no associated thrombogenicity, sterility, a long shelf life, and easy preparation/administration [49].

Platelet microparticles are microvesicles of platelet membranes and, like intact platelets, they have procoagulant activity, adhering to the subendothelium and enhancing platelet adhesion [50]. Infusible platelet membranes (IPMs) are usually produced from outdated human platelets. The source platelets are fragmented and lyophilized and can be stored for up to 2 years. The platelet membranes still express some blood group and/or platelet antigens, but they appear to have resistance to immune destruction [49]. IPMs have gone through phase 1 and 2 clinical trials in which the phase 2 trials included bleeding refractory thrombocytopenic patients, with bleeding showing some improvement [50].

Attempts at developing synthetic platelets have also investigated non-platelet-derived products. RBCs with surface-bound fibrinogen or with surface-bound Arg–Gly–Asp (RGD) peptides have been tested. Fibrinogen is the main ligand that crosslinks activated platelets to form platelet aggregates. This occurs when an RGD sequence within fibrinogen attaches to platelet integrin on glycoprotein GPIIb–IIIa. Since fibrinogen has more than one RGD sequence, activated platelets will be bridged to form platelet aggregates. Over the past years, in vitro studies have shown that RBCs bearing fibrinogen or RGD sequences may cause platelets to aggregate, but in most cases, in vivo experiments have failed to show hemostatic efficacy [50,51].

Fibrinogen-coated albumin microcapsules/microspheres (FAMs) are another synthetic non-platelet-derived product being investigated. FAM preparations are made of human fibrinogen bound to the surface of human albumin microcapsules/microspheres. Thrombospheres (Hemosphere, Irvine, CA, USA) enhance platelet aggregation; however, they do not themselves activate the platelets, so there has not been any evidence of thrombogenicity [52]. It is currently being evaluated in clinical trials. A similar product, Synthocytes (Andaris Group Ltd., Nottingham, UK), a 3.5–4.5 μm-diameter FAM, is also in clinical trials [49,50].

There has also been interest in developing a liposome-based platelet substitute. Since the initiation of lipid vesicle research in the 1960s, liposomes have remained an essential technology in the drug delivery field because of their potential to carry hydrophobic/hydrophilic molecules and appropriate time to circulate in vivo [53]. Plateletsomes, liposomes containing a minimum of 15 different platelets membrane proteins (i.e., GPIb and GPIIb-IIIa), were first investigated in thrombocytopenic rats which showed a decrease in bleeding time of 42% [54]. Similar products were also manufactured using recombinant glycoproteins such as rGPIbα [54,55]. Following this, platelet substitutes were synthesized with peptide ligands instead of whole proteins. Common peptide ligands used included collagen-binding peptide (CBP: [GPO]7) and von Willebrand factor (vWF)-binding peptide (VBP: TRYLRIHPQSQVHQI) to induce platelet adhesion and fibrinogen-mimetic peptides (RGD and H12 peptides) to mimic platelet aggregation [53].

Polymeric nanoparticles have also been under development as a synthetic platelet substitute. During one of the first studies of synthetic polymer-based platelet substitutes, Bertram et al. [56] manufactured a hemostatic nanoparticle made of a poly (lactic-co-glycolic acid)-poly-L-lysine (PLGA-PLL) copolymer conjugated to PEG arms terminated with RGD peptides which showed an efficient hemostatic effect in a rat model [53]. Meanwhile, Okamura et al. [47] developed biodegradable nanosheets that are disk-shaped which provide a large contact area for targeting sites, instead of the conventional small contact area in spherical carriers. They conjugated an H12 peptide to the surface of the nanosheets made of poly (D,L-lactide-co-glycolide) (PLGA). These nanosheets interacted with the adhered platelets on the collagen surface and revealed the formation of a platelet thrombus in a two-dimensional spreading fashion. Gao et al. [57] developed a hemostatic agent using polymer peptide interfusion (HAPPI), in which a hyaluronic acid is conjugated to a collagen-binding peptide and a vWF–binding peptide. HAPPI selectively bound to activated platelets and promoted platelet accumulation at the wound site, which significantly reduced bleeding in a mouse model. SynthoPlate is a nanoparticle product that has been designed to mimic platelet adhesion and aggregation. During studies, it did not affect resting platelets but amplified the aggregation of active platelets, which resulted in enhanced fibrin generation. It reduced bleeding time in mouse models. It also demonstrated an appropriate circulation time. It is being evaluated in clinical trials and is also being studied to be applied as a lyophilized powder at the point of injury to control hemorrhages [48,58].

Although the development of a successful FDA-approved, synthetic platelet substitute holds promise, achievement remains elusive given the challenges of developing a product that is effective (i.e., hemostatic), stable (in storage and in vivo), and non-immunogenic, as noted above in this section. Toward that end, the ex vivo engineering of platelets, essentially the manufacture of platelets through human-induced pluripotent stem cell technology to derive immortalized megakaryocyte progenitor cell lines, perhaps holds the key to true success in alleviating chronic platelet supply shortages [59]. Furthermore, recent investigation into platelet clearance mechanisms, including platelet apoptosis and peptide ligand binding of plasma vWF to the ligand-binding domain of platelet GPIbα, among others, may lead to novel strategies to extend the shelf-life of platelets stored at RT or to enable cold storage which also could contribute to the alleviation of platelet supply shortages [60].

## 8. Paid Platelet Donors

The US moved to an all-volunteer blood donor system in 1970 as a means to reduce transmissible disease risk, mainly non-A, non-B viral hepatitis at a time prior to the human immunodeficiency virus (HIV) epidemic [61]. Yet, because there has been a growing concern that strict reliance on a voluntary blood donor system has not been robust enough to meet the increasing demand for blood products, some authors have proposed the remuneration of donors to ensure a safe and available blood supply [62]. Stubbs et al. [21] notably made the point that donor remuneration would have the potential to attract younger donors. In regard to product safety concerns, especially transmissible disease risks, these authors and others point to the commercial plasma industry as an example of safety in using paid donors to maintain a constant supply, noting enhanced donor screening, repeat donors, and PR technology as measures of safety [21,63]. However, the idea of financial incentives for encouraging blood product donation is not without controversy, and this controversy encompasses both safety and ethics. Dodd et al. [64] cite negative effects on donor altruism while also arguing that the platelet supply can be grown independently of paid donors and that the fundamental challenge is the short shelf life of platelet products rather than the lack of donors. Meanwhile, the WHO takes a hardline stance in advocating for the use of voluntary nonremunerated blood donations [65]. It argues that remuneration threatens blood product safety and increases the vulnerability of disadvantaged populations as well as the potential to reduce voluntary donations. Yet, remuneration is poorly defined, such that even gift cards or time off from work that may be given by US donor collection centers and organizations and that are not considered payment by the FDA do not meet the “voluntary” standard of the WHO [65]. Critics note that remuneration can cause socioeconomically vulnerable people to overlook the risks associated with blood donation, as they may see paid donation as a necessary method of obtaining money. This population of donors may also not fully understand the risks, including the potential long-term effects of frequent donation, to themselves (e.g., iron deficiency, reduced plasma proteins), due to limited health literacy [65]. Therefore, the informed consent process may be inadequately understood. A potential area of future research would be to study the level of comprehension of informed consent between voluntary and remunerated blood product donor groups.

## 9. Conclusions

Current U.S. platelet transfusion practices are evolving. Table 1 summarizes key platelet transfusion innovations on a timeline. Lower-dose (2.0–2.5 × 10^11^ platelets/unit) and alternative products (pathogen-reduced, cold-stored, or freeze-dried platelets) are increasingly being considered for use. Innovations in storage and processing enhance safety but require yield trade-offs. Emerging options like synthetic substitutes and revived whole-blood-derived platelets may address supply challenges. The key unresolved issues include HLA alloimmunization risks and optimal pediatric dosing. Moving forward, integrating evidence-based dosing with technological advances and diversified inventories will be crucial for balancing safety, efficacy, and supply stability in transfusion medicine.

## Figures and Tables

**Table 1:** Timeline of platelet transfusion innovations in the United States.

Timeline	Platelet Transfusion Innovations in the United States
1960s	The role of platelet transfusions for management of bleeding in cancer patients is first recognized [1].
1970s	Apheresis technology for the collection of platelets is developed. Movement toward an all-volunteer blood donor system begins [62].
1980s–present	The United States becomes the leading nation in platelet transfusions per capita [1].
1990s–2000s	Bacterial contamination rates of platelets are increasingly recognized [66].
2004	The Association for the Advancement of Blood and Biotherapies (formerly the American Association of Blood Banks) implements its Standards to limit and detect bacterial contamination in all platelet components [66].
2010	A platelet additive solution for apheresis platelet storage is introduced [67].
2014	A pathogen inactivation system for platelets is introduced [68].
2019	The Food and Drug Administration Guidance on bacterial risk control strategies to enhance the safety and availability of platelets for transfusion is published (updated December 2020) [5].
2020s	Increasing challenges of platelet collection shortages lead to calls for apheresis donor remuneration [62].
2020s–present	There is continued advancement into the development of platelet alternative technologies [57,58]
2023	The Food and Drug Administration Guidance for the manufacture and labeling of cold-stored platelets is published [40].
